# Understanding the Influences of COPD Patient’s Capability on the Uptake of Pulmonary Rehabilitation in the UK Through an Inclusive Design Approach

**DOI:** 10.2147/COPD.S305145

**Published:** 2021-06-16

**Authors:** Yuanyuan Liu, Terry Dickerson, Frances Early, Jonathan Fuld, Chen Jiang, P John Clarkson

**Affiliations:** 1 Department of Industrial Design, School of Mechanical Engineering & Automation, Beihang University, 37 Xueyuan Road, Haidian District, Beijing, 100191, China; 2 Engineering Design Centre, Department of Engineering, University of Cambridge, Cambridge, CB2 1PZ, UK; 3 Centre for Self Management Support, Cambridge University Hospitals NHS Foundation Trust, Cambridge Biomedical Campus Department of Respiratory Medicine, Cambridge University Hospitals NHS Foundation Trust, Cambridge, CB2 0QQ, UK; 4 Department of Clinical Neurosciences, University of Cambridge, Clifford Allbutt Building, Cambridge Biomedical Campus, Cambridge, CB2 0AH, UK

**Keywords:** COPD, pulmonary rehabilitation, healthcare access, care journey, Inclusive Design, capability

## Abstract

**Background:**

Pulmonary rehabilitation (PR) is recommended for patients with COPD to improve their symptoms and quality of life. However, in the UK, only one in ten of those who need PR receive it and this might be inaccessible to people with disabilities. This study aims to inform improvements to PR service by identifying barriers to the uptake of PR in the COPD care journey in relation to patients’ capabilities that can affect their access to PR.

**Methods:**

An Inclusive Design approach with mixed methods was undertaken. Firstly, patients and healthcare professionals were interviewed to gather insight into their experiences of COPD care and map patients’ care journey. Secondly, an Exclusion Calculator was used to estimate service demand on patients’ capability and the proportion of population excluded from the service. Thirdly, a framework analysis was applied to guide data analysis to identify the challenges of accessing PR. Finally, proposed recommendations were refined with patients and healthcare professionals.

**Results:**

The overall capability-related exclusion number was very high (62.5%), and exclusion caused by limited mobility was the highest (50%) among the interviewees and even higher based on the population database. This suggests the importance of considering COPD patients’ capability-related needs to improve their access to care. Capability-related challenges for patients accessing PR such as poor mobility to transport and low vision impairing ability to read inhaler instructions were identified, as well as non-capability-related challenges such as patients’ perception about COPD and inability to access proper information. Recommendations were proposed to help patients to self-manage their COPD and access to PR.

**Conclusion:**

Lack of attention to COPD patients’ capability level in the delivery of PR may affect its uptake. Considering the capability-related needs of COPD patients and providing patients with reassurance, information, and support on their care journey could improve the uptake of PR.

## Introduction

Chronic Obstructive Pulmonary Disease (COPD) is a progressive life-threatening lung disease that causes people breathlessness, exercise incapacity, frequent infections and hospitalization. According to the World Health Organization’s (WHO) estimate, over 3 million people died from COPD in 2005, which corresponded to 5% of all death worldwide.^1^ The prevalence of COPD is likely to increase regionally and globally in the coming years.^2^ In the UK, approximately 1.2 million people were living with diagnosed COPD and 2 million people have undiagnosed COPD.^3^ The annual direct healthcare cost of COPD in England has been estimated to increase from £1.5 billion in 2011 to £2.32 billion in 2030, mostly relating to hospital admissions.^4^ The UK National Institute for Health and Care Excellence (NICE) recommends pulmonary rehabilitation (PR), providing supervised exercise and education, as an evidence-based non-pharmacological treatment for COPD patients.^5^ It leads to clinically significant improvements in symptoms, exercise capacity and health-related quality of life,^6^ and results in fewer and shorter hospital attendance and readmission.^7^,^8^ In contrast to the treatment and management of COPD from the biology of the disease,^9^,^10^ PR focuses on fostering self-management skills for patients,^11^ and preventing their condition from deteriorating. British Thoracic Society guidelines suggest PR can be offered to patients functionally disabled by COPD.^6^

Despite evidence-based guidelines recommending PR, it is still underutilized in practice worldwide. For instance, the UK National COPD Audit Program (2016) estimated that the number of COPD patients eligible for PR in England and Wales in 2013/14 was 446,000. However, only 68,000 patients were referred to PR programs during that period. Of those, only 69% attended an initial assessment.^12^ More recently, the National Asthma and Chronic Obstructive Pulmonary Disease Audit Programme (2020) reported in PR clinical audit 2019 that only 58.0% of patients with stable COPD started PR within 90 days of receipt of referral (with an average waiting time of 78 days), which was far below the national goal of 85.0%.^13^ The Audit highlighted the importance of ensuring patients start PR within 90 days. The low rates of referral and uptake impede access to this cost-effective PR, so it is urgent to identify and address barriers that prevent patient access. The Audit recommended reviewing PR referral pathways, healthcare professional training, information for patients and referrers, and patient access barriers. It also stated that COPD treatment might not be equally accessible to people with disabilities.^14^

Several studies have been conducted to understand barriers that affect access to PR.^15–26^ Referral to PR can be influenced by complicated referral processes, lack of knowledge or information about PR, and unclear roles and responsibilities among healthcare professionals (HCPs) concerning referral. Uptake rates can be affected by the patients’ beliefs about the benefits of PR, timing, transport and even geographic distance to a program as well as the quality of the HCP’s conversation with patients about PR. Most of these identified barriers pertain to context or environment, people’s knowledge and patients’ and clinicians’ beliefs.^27^ Few studies have investigated the association between patients’ own physical and cognitive capabilities (including mobility, dexterity, reach and stretch, vision, hearing, thinking and communication) and their ability to access PR, which may affect the implementation of a PR service.

Any healthcare service makes demands on patients, for which patients have to have sufficient capabilities in order to respond to these demands and access the service. Capability in this research context mean people’s abilities to access a PR service. There are mainly two factors: age-related change and condition-related change in people’s capability. For patients with COPD, a large proportion are older people whose COPD condition, along with the ageing process, may significantly impact their capability.^3^ COPD patients are more likely to be frail, weak and have reduced physical activity levels.^26^ If the demands of accessing the PR service exceed COPD patients’ capabilities, they may be excluded from the PR service. For example, when the PR venue is far away from a patient’s home and the patient’s mobility is limited, the patient may not be able to get to the venue. In this case, the patient is likely to decline or not accept the program. Hence, it is essential to consider patients’ capabilities in order to improve access to and use of PR services.

Inclusive Design aims to ensure that the demand made on an individual in a given environment does not exceed their capability to respond so that the product or service is accessible to as many people as possible.^28^ It has been widely used in improving the accessibility of buildings and public transport,^29–31^ and recently has been used to improve the accessibility of healthcare services including secondary care and back pain.^32^,^33^ There is a high possibility that Inclusive Design could improve COPD patients’ access PR service and thus improve the utilization of the services.

Therefore, this study aimed to use Inclusive Design methods to identify capability-related challenges along COPD patients’ care journey while accessing PR and propose ways to increase uptake and attendance to PR services.

## Methods

### Overview

**Figure 1:**
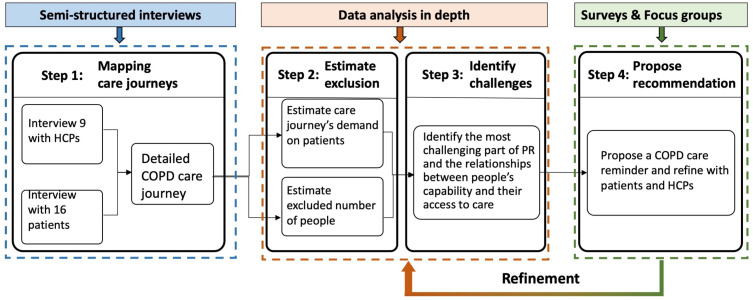
Study design.

Figure 1 illustrated the four-steps of this Inclusive Design approach: i) Step 1: Mapping care journeys, ii) Step 2: Estimate exclusion, iii) Step 3: Identify challenges, and iv) Step 4: Propose recommendations. Specifically, in Step 1 semi-structured interviews were conducted with HCPs and patients to gather insight into their experiences and produce a hierarchical task analysis^34^,^35^ of the COPD care journeys. Step 2 estimated the service exclusion: the demand of every task on patients’ capabilities was rated using pre-defined scales, and the proportion of the UK population excluded from the service was estimated by an Exclusion Calculator.^36^ Step 3 identified the challenges of the PR service: a framework analysis^37^ guided further data analysis of the interviews. Step 4 proposed recommendations that would help patients manage their COPD care, informed by addressing the challenges identified in step 3 and refined through a survey and focus groups. Here we focused on presenting data analysis and explained how we consider the research bias. More detailed explanation of methods can be found in the published protocol of this study,^38^ which introduced the detailed Inclusive Design approach, study design, sampling and recruitment, ethics and data protection.

Ethical approval for this study was provided by the Cambridge Central Research Ethics Committee (17/EE/0136). The study was conducted in accordance with the Declaration of Helsinki. A participant information sheet was provided to each participant to inform them of the research, data collection, and their rights. Participants could withdraw their participation at any point, and all data were kept anonymous. The consent form was signed before their participation.

### Data Analysis

#### Step 1: Mapping Care Journeys

In order to map patients’ COPD care journey, semi-structured interviews with HCPs and patients were conducted to gather insight into their experiences and perceptions of the COPD care pathway and care journeys respectively. It is important to clarify that this research only focuses on patients’ access to PR services. Patient’s ability to do exercises and take part in PR once they accessed the service and the PR program itself were not the focus of this research.

All interviews were audio-recorded and transcribed verbatim. Transcriptions and field notes were managed and analysed using NVivo 12, a software support tool for qualitative and mixed methods research, which is designed to help organise, analyse and find insights in unstructured or qualitative data.^39^ The activities that HCPs and patients would do within primary care were coded based on the care pathway’s stages (Diagnosis, Review, Referral, Assessment, and PR program) in NVivo. Meanwhile, the understanding of HCPs’ care pathways also helped map the patient care journey, for example, the consultation and referral process in the patient care journey can be inferred from HCPs’ care pathways. Based on the coding data of patients and HCPs’ care pathways, the representative activities that most patients experiences along their care journeys of receiving COPD treatment was summarized.

#### Step 2: Estimate Exclusion

Step 2 contains two parts: a) estimate care journey’s demand on patients, b) estimate the excluded number of people.

##### Estimate Care Journey’s Demand on Patients

It was important to define the scope and estimate a representative care journey’s demands on patients’ capabilities, as different patients may experience various care journeys and it was impossible to cover all possibilities. Two “aided tools” of Inclusive Design, a persona (fictional characters that are based on real information)^40^ together with a scenario (that describes the stories and context of how people experience services) were used to define a representative care journey according to Mrs. Smith’s real stories (Table 1) about accessing PR services. The name of Mrs. Smith is not the real name due to the data confidentiality and serves only the purposes of referring. Some detailed descriptions from other similar patients were used to complement the scenario in order to reveal the potential challenges that prevent patients from accessing PR services.

**Table 1:** The Scenario of a Persona: Mrs. Smith Accessing PR Service

Persona	Mrs Smith, a 65-years-old, retired office worker, lives with her husband in a village. She is a central hub of her family. She has a son and a daughter who is disabled and lives a mile away from her home. Mrs Smith is active, she likes shopping and gardening, but she recently felt breathlessness and could not function as before. She felt quite upset and sometimes found herself muddled in communication with friends. She was diagnosed with COPD one and a half years ago likely because of smoking. Her husband has chronic back pain.
Service name	Pulmonary rehabilitation (PR) for patients with COPD in the community
User tasks being assessed	The service’s demand on patients’ capability when they access PR services, mainly including representative tasks that patients are likely to be involved in: Diagnosis, Review, Referral, Assessment, and PR.
Scope	**In scope**: This research only identifies the COPD care pathway (journey) that related to patients’ access to PR services. **Out of scope**: Patient’s ability to do exercises and take part in PR once they have accessed the service, and the PR program were not the focus of this research. Tasks that are similar to older people’s everyday activities such as make a call and open a letter are not assessed, and tasks where assistance is available are also not assessed. **Starting point**: The patient experience some breathing issues and books a GP appointment.
User scenario based on true stories	Here is some necessary information for the assessment: Distance from Home to GP practice: 1 km; Distance between car park and GP practice: 20–50 m. Distance from Home to Assessment/PR class (the same place as assessment): 30km; Distance between car park and Assessment/PR class: 300m. PR program: 8 weeks program, which contains 16 sessions and two sessions a week. Each session involves about an hour for exercise and half an hour for education, and a tea break between exercise and education. **Diagnosis**: Mrs Smith had a sign of asthma and became breathlessness, so she decided to see her GP. She made a GP appointment by telephone, and she needed to put on her hearing aid as her left ear is impaired. Although the GP practice was not far from her home, Mrs Smith went there by car. She parked and signed in through the self-check counter. The GP asked her symptoms and referred her to further check by the practice nurse. The nurse asked her to do some tests such as the spirometry test and measured her blood pressure to help the diagnosis. Then, she returned home and waited for the further information. About five days later, she received a letter from the GP practice which informed her she had COPD. She was shocked because she had no idea what COPD was, and there was no more information offered to her in the letter. She was particularly distraught because she has a history of depression. It took her about two weeks to calm down before she phoned the nurse for further consultation. However, the nurse in the GP practice she called did not have much knowledge about COPD care, and she just received two inhalers (medications) for the treatment and was advised to quit smoking without further help. At home, she found it difficult to read the instruction of one inhaler, because the font size was too small. Also, she found her vision declined, and her eye doctor suggested she change her glasses. She thought the inhaler had side effects and could lead to cataracts. **Review**: Ten months after the diagnosis, Mrs Smith received an invitation letter for a COPD annual review. As usual, she went to the review by car, and her husband accompanied her as they were looking for more information to help her condition. They arrived a little bit late, so she had to sign in through the reception. The nurse (who was not the same as the one who did the diagnosis) did the annual check, reviewed the medication, assessed her COPD condition. Mrs Smith wanted to keep active, and she asked the nurse whether she could go swimming. The nurse asked Mrs Smith, “why didn’t you go for PR?”, and the nurse suggested she try the PR which contained exercise and education. She felt a bit sad as she was not offered PR when she was diagnosed, and the nurse seemed not to know her and her condition. She wondered whether the nurse could tell her a bit more information, but the nurse did not have a PR leaflet available. Regardless of that, Mr Smith encouraged his wife to have a try, so she decided to have a go. **Referral**: About one week later, Mrs Smith received the referral letter, which included an appointment for assessment and a leaflet about PR. She was quite happy about the opportunity to do some exercise, so she decided to go.
User scenario based on true stories	Assessment: The assessment venue was quite far away, so she asked her husband to drive. There were no detailed navigation instructions like a map in the invitation letter, so they had to plan the transport route by entering the postcode from the invitation letter into the car’s GPS system. However, it was not easy to find the assessment venue from the car park, due to the limited mobility. Mrs Smith stayed in the car while her husband wandered around to find the reception. Then her husband came back to pick up Mrs Smith once he had sorted it out. As a result, it took them a while to find the front reception. The physiotherapist did the assessment for her, which included questionnaires, a review of her medication, and a walking test, etc. Mrs Smith worried that she could not make two times a week as the class was very early in the morning, so she tended to say No. Luckily, the physiotherapist who was very considerate, tailored the class schedule for Mrs Smith, ie, one session per week instead of two sessions per week. She was very pleased with the customised plan, so she decided to attend the PR class. However, due to the limited space in the PR class, she had to wait about two months to start her class. During the waiting period, Mrs Smith had a difficult time since her condition had become worse. She often felt depressed because she was not able to do daily activities as she used to. PR: The PR class starts at 9 am and finishes at 11 am, so Mrs Smith had to get up quite early. She drove by herself as her husband’s back pain had worsened and she already knew where the PR class was. She arrived at the car park of the PR class and walked to the reception. However, there were about 300 metres away from the car park to the classroom. She had to stop and rest several times on the way because 50 metres is the furthest distance she can walk without needing to stop due to discomfort. When she arrived at the classroom, she felt a bit tired, which prevented her from effectively taking part in the exercises. After attending a few times, Mrs Smith noticed that some people had the transport service, so she requested the transport service. However, the PR provider rejected her application for the transport service due to the limitation of the service, which is only available to people who live very far and could not drive. As a consequence, she decided to quit the PR service as she did not want to be a burden to her family (to get there) but going by herself was too onerous.

There were two reasons why the care journey of Mrs. Smith and people similar to her was selected as a prototype to assess the PR service’s demands on patients. Firstly, Mrs Smith had accepted a PR offer and experienced all five stages of the COPD care. Secondly, Mrs. Smith’s situation could be used to gain insights into why some people with COPD are not referred or decline since they cannot access the PR services. The main difference in care journeys between people who are not referred or decline and people who accept PR is that those people who are not referred or decline only experience part of the primary COPD care journey, signaling challenges along the COPD care journey that prevent them from accessing PR services. Therefore, Mrs. Smith’s case would be a representative care journey to estimate the capability demand and understand the potential challenges along patients’ care journey.

According to Mrs. Smith’s scenario, the way (option) Mrs. Smith, and similar patients, conducted each activity was defined and further specified as tasks for assessment (Table 2). For example, there are several ways for patients to undertake the activity “Transport” including driving, walk, bus and by other means, while Mrs. Smith chose to walk. Then the demand on every task/activity was assessed in accord with the pre-defined scales (Figure S1) by the main researcher and further checked by an expert of Inclusive Design.^41–43^

**Table 2:** The Specified Tasks Based on Mrs. Smith’s Care Journey Accessing PR Service

Stage	Diagnosis										
Task No.	1	2	3	4			5	6	7	8	9
Task name	Walk	Describe symptoms	Wait 20 mins	Go to the nurse’s office			Describe symptoms	Read letter	Open the package (inhalers)	Read the instructions (inhalers)	Use the inhalers
Stage	**Review**									**Referral**	
Task No.	10	11	12				13	14		15	
Task name	Read the letter	Walk	Answer questions for review				Offered the PR class	Request information about PR		Read the letter	
Stage	**Assessment**										
Task No.	16	17	18	19		20	21	22	23	24	25
Task name	Ask her husband to help drive	Read the instructions for travel in referral letter	Walk and stop to rest after every 50m of walking (in total 300m distance)	Provide invitation and medication to be checked		Fill in question-aires	Walking test	Obtain more info about PR	Request to tailor the class schedule	Obtain the tailored class schedule	Agree and wait to attend
Stage	**PR**										
Task No.	26		27		28			29		30	
Task name	Drive to the same place of assessment		Walk and stop 4–5 times to rest (in total 300m)		Find out about the transport service			Request transport service		Decide to quit	

##### Estimate the Excluded Number of People

There were two ways to estimate the excluded number of people who would not be able to access PR: i) using the British population database within the current “Exclusion Calculator” to measure exclusion from access to PR services within the general population (of which only some will have COPD); ii) screening the capability data of people with COPD from the available British population database and estimate the exclusion of people with COPD from access to PR services.

**Figure 2:**
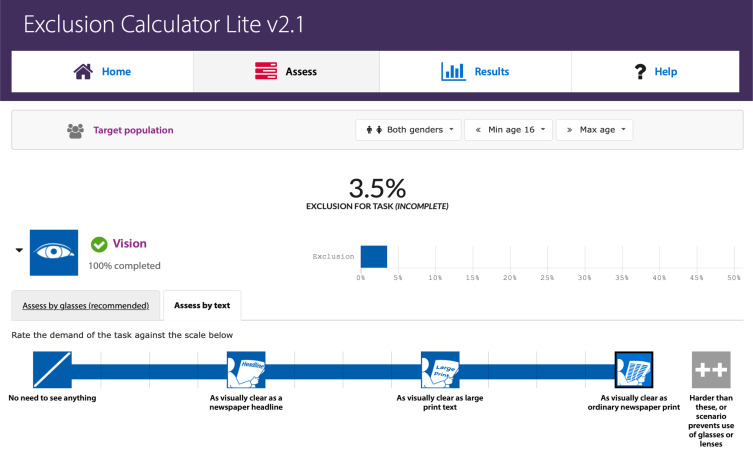
Calculating exclusion (ie, the vision exclusion for the task “fill in questionnaire”).

Figure 2 shows an example of measuring the vision exclusion for the task “fill in questionnaire”. The demand of vision capability is similar to read the original printed newspaper, so it was rated as scale 12 and about 3.5% British population may not complete that task. By inputting the estimated demands (rated scale) of each task along the COPD care journey, the “Exclusion Calculator” can estimate the number of people within the UK general population excluded from accessing PR (in every task and on the whole care journey). Similar to assess the scale, all the calculation was completed by the main researcher and checked by an expert of Inclusive Design.

In terms of the second way of estimating the exclusion, it is necessary to review the available population data. The original population data (sample size n=7618) was taken from the Disability Follow-up to the Family Resources Survey (DFS).^41^ The survey was based on the adult population in Great Britain living in private households, which was 43.3 million people at the time of the survey. Among these participants, 126 participants had COPD and 962 participants self-reported that they had a respiratory issue, and it is likely that a significant number of these people could benefit from PR. A senior respiratory specialist from Cambridge University Hospitals filtered the data from the whole population data to ensure rationality. It was considered meaningful to screen those people’s capability data and measure the exclusion of people with COPD (Group 1), those who have a respiratory issue (Group 2) and the 16 interviewed patients (Group 3) when they access the PR service.

#### Step 3: Identify Challenges

The challenges in accessing PR were identified in two ways: further analyzing the interview data with HCPs and patients; and consulting the data about the demands on patients of the PR care journey and the excluded number of people. Framework analysis,^37^ which enables themes to be developed inductively from the experiences and views of participants, was used to structure the data analysis for interview transcripts. All the raw data was familiar and managed in NVivo12, and then the initial codes and categories were created based on the key words of research questions and five participants’ transcripts which were selected to cover different roles of participants, consisting of patients who accepted, declined and referred PR offer and HCPs including a GP and a physiotherapist. The initial codes and their definitions were defined by the main researcher and were further checked by another two researchers to minimize bias and ensure comprehensibility. Using this initial coding frame, all the interview transcripts were coded in NVivo12. Inductive coding was used in order to incorporate emergent codes, for example, a new code “after PR” was added. The two previous researchers were also involved in reviewing the refining process to ensure the final themes were agreed. The potential needs of patients discerned from the interpretation of patients and HCPs’ challenges, which laid the foundation for proposing recommendations. Expectations and needs of patients from an HCP’s perspective also were also considered in making recommendations.

#### Step 4: Propose Recommendations

**Figure 3:**
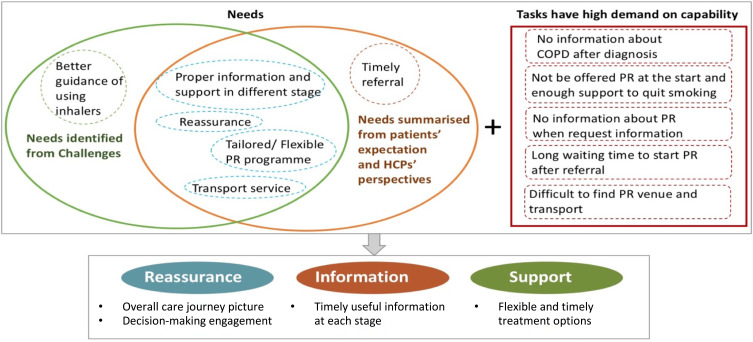
Patient’s needs to better access PR.

Integrating the summarized patients’ needs and demand as well as exclusion data, three themes were suggested to increase patients’ access to PR (Figure 3). Based on the themes, the initial recommendations, which aim to address patients’ challenges and meeting their needs when accessing PR were proposed. To evaluate the recommendations, two focus groups with patients from Breathe Easy Cambridge Support Group and a survey with HCPs (n=10). The focus group invitation was given to patients one month before the discussion during their monthly group meeting. The HCP survey was disseminated after the East of England PR meeting at Cambridge University Hospitals, and was completed anonymously by PR service managers, physiotherapists and respiratory nurses. After the recommendations was refined based on the comments of the two focus groups and survey, one more focus group with patients was organized to further check the refinements. Finally, the comments collected from the focus groups and survey were summarized and all the refined recommendations were put into a set of 7 cards named “Your COPD Care Reminder”.

### Research Bias

To reduce the chances of acquiescence bias when designing research, the interviews and focus groups were carefully designed and reviewed by experts including healthcare professionals as well as researchers, and patients to ensure the questions were short and clear, and the answer choices were short and concise. The research protocol^38^ was reviewed by the Research Advisory Committee (RAC) of Cambridge University Hospitals and advice was received from the members of the Patient and Public Involvement (PPI) panel, Cambridge University Hospitals (CUH, an NHS Foundation Trust) before being sent to the ethics committee. To reduce the risk of confirmation bias during data analysis, the work was supervised by a senior researcher of the EDC. A second researcher from Cambridge University Hospitals independently analyzed a sample of the data at each stage of the analysis. The primary and secondary analysts compared the results and resolved any discrepancies. Should any discrepancies not have been resolved, the senior supervising researcher would have played the role of adjudicator. In the steps of refining the recommendations, acquiescence bias may also appear as the HCPs and patients may view the researcher as an expert. An anonymous survey with HCPs in step 4 (propose recommendations) enabled them to evaluate the recommendation with less biased feedback. Having focus groups with some patients twice may also help to notice if there is an acquiescence bias, although it could not be avoided.

## Results

### Mapping Care Journey (Step 1)

In total, 9 HCPs were interviewed, including GPs (n=2), practice nurses (n=2), physiotherapists (n=3), PR service managers (n=1) and healthcare commissioners (n=1). 16 patients with COPD were interviewed: 10 patients who had accepted a PR offer (including 2 patients who had declined a second PR offer and 1 patient who had not been referred PR again), 4 patients who had not been referred to PR, and 2 patients who had declined their first and only PR offer. The detailed demographical data of participants can be found in Table 3.

**Table 3:** The Demographic Data of Participants

Category	PR Programme	Gender	Age Group	Capability
Step 1 Interview patients	Accepted PR offer (n=9) Declined PR offer (n=5) Never referred (n=2)	F=6 M=10	50–64 (n=2) 60–74 (n=8) >75 (n=6)	Ensure a full range of capability loss including Hearing, Vision, Mobility, Dexterity, Reach and Stretch, Communication, Thinking, is covered by the samples
Step 4 Focus group with patients	Mixed 1st Focus group (n=4) 2nd Focus group (n=5) 3rd Focus group (n=8)	Mixed	N/a	N/a
**Category**	**HCP’s Role**	**Gender**	**Years of Working**	**Understanding of PR**
Step 1 Interview HCPs	GP (n=2) Practice nurses (n=2) Physiotherapists (n=2+2) PR service manager (n=2) Commissioner (n=1) Note: Two service managers also physiotherapists	F=8 M=1	>10 years (n=5) 3–5 years (n=4)	Familiar with the process of diagnosis, annual review, referral, assessment for PR, and PR (part/all of the process) Familiar with the process of designing or supporting the COPD care pathway
Step 2 Surveywith HCPs	Mixed Service manager Nurses physiotherapists	Mixed	N/a	

The main activities within primary care for most patients with COPD are summarized in Table 4. There are several different possible routes for patients to access PR services and Table 4 lists most of the representative possibilities described in the interviews. The main stages in the UK National Health Service (NHS) primary care pathway for COPD rehabilitation are: Diagnosis, Annual review, Referral for PR, Assessment for PR and PR program. Taking the activities of the diagnosis stage as an example, patients may need to go through making an appointment with their GP, transport to appointment, consulting with their GP during the appointment, being referred for further assessment by practice nurse, being assessed by nurse, being informed of diagnosis, receiving self-management treatments, and deciding to take up PR (if offered). There are many options available for these activities, for instance, making an appointment with GP can be done by telephone, going into the surgery, by others and by Internet. The tasks for any one patient accessing PR will depend on the options they choose. It is worth noting that different patients may experience different tasks along their care journeys, and it is impossible to cover all the possibilities.

**Table 4:** Patient’s Potential Care Journeys to Access PR

Stages		Diagnosis																	
Location		Home ——> GP Practice ——> Home																	
Activities		Make an Appointment with GP			Transport		Consult GP Appointment		Referred to Further Check		Checked by Nurse			Informed Diagnosis		Receive Self-Management Treatments		Decide to Take PR (If Offered)	
Options	**1**	By telephone			Drive by oneself		N/a		Referred to practice nurse		N/a			By face-to-face		Medications (inhalers)		Not offered	
	**2**	Drop in			Driven by family/friends				Referred to secondary care					By call		Medications (emergency pack)		Offered but declined	
	**3**	By others			On foot				…					By letter		Pulmonary Rehabilitation (PR)		Offered and accepted	
	**4**	By Internet			By bus+ walking				…					…		Quit smoking		…	
	**5**	…			…				…					…		…		…	
**Stages**		**Review**														**Referral**			
**Location**		**Home——> GP Practice ——> Home**														**Home**			
**Activities**		**Receive Invitation to Review**		**Transport**			**Checked by Nurse**			**Discuss Care Plan**			**Make a Decision (PR)**			**Receive Inform for Referral**		**Make a Decision (PR)**	
Options	**1**	By letter		Drive by oneself			N/a			Offered PR			Attend			By letter		Attend	
	**2**	By call		Driven by family/friends						Received updated medication			Not attend			By call		Not attend	
	**3**	By message		On foot						…			…			…		…	
	**4**	…		By bus+ walk						…			…			…		…	
	**5**	…		…						…			…			…		…	
**Stages**		**Assessment**										**PR**							
**Location**		**Home ——> Assessment avenue ——> Home**										**Home ——> PR class ——> Home**							
**Activities**		**Plan to Get to Car Parking of the Assessment**	**Plan to Get from Car Parking to Front Reception**			**Transport**		**Assessment Test**		**Make a Decision (PR)**		**Plan How to Get to PR Class and Go Back**			**Transport**		**Request for Transport Service**		**Continue to Attend PR**
Options	**1**	By letter	By asking passerby			Drive+ walk		N/a		Attend		Ask family/friends			Drive + walk		Approved	Attend	
	**2**	Ask friends	By calling reception			Family/friends drive				Not Attend		By memory			Transport service		Rejected	Quit	
	**3**	By internet	By following carer			Bus				…		By satellite navigate			Walk		…	…	
	**4**	By satellite navigate	…			Walk				…		…			Bus		…	…	
	**5**	…	…			…				…		…			…		…	…	

### Estimate Exclusion (Step 2)

#### Estimate Care Journey’s Demand on Patients

The care journey’s demand on every task of PR service based on Mrs. Smith’s scenario was estimated (see Table S1). Generally, a higher demand on people’s capabilities leads to higher scale ratings, and the symbol “>” (off scale) which means it is excessive for a mainstream service for people to access. There were seven times rated “>” on patients’ capabilities, including four times rated “>” on patients’ vision, twice rated “>” on patients’ literacy, one time rated “>” patients’ memory. For example, the capability vision and literacy rated “>” of task 6 “read letter (Informed diagnosis by letter)” due to lack of information about COPD for patients to read in the diagnosis letter. Although the rated score for walking capability in task 18, task 27 and task 30 was rated only “10” (marked in bold), it was still most likely to beyond the walking capability of patients with COPD. The last column of the table shows the overall demands of all the tasks.

#### Estimate the Excluded Number of People

Table 5 shows the results of the first way to estimate the exclusion data. Based on the PR service demands on patients’ capability, about 15.6% of British people who experience a similar scenario to Mrs. Smith could be excluded from the PR service.

**Table 5:** The Exclusion of Every Stage (Based on Table 1 Mrs. Smith’s Scenario)

Stage	Diagnosis	Review	Referral	Assessment	PR	Overall Exclusion
Exclusion (%)	12.6	12.3	8.9	14.8	15.3	**15.6**
Vision only (%)	4.1	4.1	4.1	4.1	4.1	4.1
Hearing only (%)	2.3	2.3	0.0	2.3	2.3	2.3
Thinking only (%)	4.6	4.6	3.0	5.2	5.0	5.3
Reach & dex only (%)	5.7	4.8	4.8	3.2	5.4	6.1
Mobility only (%)	4.7	4.7	0.0	**9.9**	**9.9**	**9.9**

**Note**: Numbers in bold correspond to the overall exclusion and the highest excluded capability.

The detailed exclusion for each task was rated (Table 6). The highest excluded task, the one that placed the highest demand on patients’ capabilities in each stage, is marked in bold. For example, the highest excluded task among those in the diagnosis stage was No. 8 “Read the instructions (inhalers)”, as the font size of the inhaler’s instruction was too small to read (smaller than the typical newspaper’s font which rated 12). In the PR stage, the highest excluded task was task No. 30 “Decide to quit (PR)”, due to the demand on patients’ capability when driving and walking to the class venue (300 m is far beyond what a person who needs rest every 50 m can manage). The last column of each stage shows the overall exclusion in that stage.

**Table 6:** The Exclusion of Every Task and Each Stage (Based on Table 1 Mrs. Smith’s Scenario)

**Stage**	**Diagnosis**											
**Task No.**	**1**	**2**	**3**	**4**	**5**		**6**		**7**	**8**	**9**	**Overall Exclusion**
Exclusion (%)	8.9	4.4	2.7	4.9	4.4		7.7		7.2	**9.4**	7.0	12.6
Vision only (%)	4.1	0.0	0.0	2.6	0.0		4.1		2.6	4.1	2.6	4.1
Hearing only (%)	1.1	2.3	1.1	0.0	2.3		0.0		0.0	0.0	0.0	2.3
Thinking only (%)	1.7	2.7	1.7	1.4	2.7		0.8		1.4	3.6	3.4	4.6
Reach & dex only (%)	0.0	0.0	0.0	0.0	0.0		4.8		4.8	4.8	2.8	5.7
Mobility only (%)	4.7	0.0	0.0	2.4	0.0		0.0		0.0	0.0	0.0	4.7
**Stage**	**Review**										**Referral**	
**Task No.**	**10**	**11**	**12**	**13**	**14**	**15**		**Overall Exclusion**			**15**	**Overall Exclusion**
Exclusion (%)	**8.9**	**8.9**	5.7	5.7	7.9	**8.9**		12.3			**8.9**	8.9
Vision only (%)	4.1	4.1	0.0	0.0	4.1	4.1		4.1			4.1	4.1
Hearing only (%)	0.0	1.1	2.3	2.3	2.3	0.0		2.3			0.0	0.0
Thinking only (%)	3.0	1.7	4.3	4.3	3.9	3.0		4.6			3.0	3.0
Reach & dex only (%)	4.8	0.0	0.0	0.0	0.0	4.8		4.8			4.8	4.8
Mobility only (%)	0.0	4.7	0.0	0.0	0.0	0.0		4.7			0.0	0.0
**Stage**	**Assessment**											
Task NO.	**16**	**17**	**18**	**19**	**20**	**21**		**22**	**23**	**24**	**25**	**Overall Exclusion**
Exclusion (%)	3.9	7.9	**12.1**	6.7	8.2	7.5		4.9	4.9	4.6	1.6	14.8
Vision only (%)	0.0	4.1	4.1	0.0	4.1	2.6		0.0	0.0	0.0	0.0	4.1
Hearing only (%)	2.3	0.0	0.0	2.3	0.0	2.3		2.3	2.3	2.3	0.0	2.3
Thinking only (%)	2.0	2.8	1.4	4.3	4.6	4.6		3.3	3.3	3.0	1.6	5.2
Reach & dex only (%)	0.0	3.2	0.0	1.6	1.6	0.0		0.0	0.0	0.0	0.0	3.2
Mobility only (%)	0.0	0.0	9.9	0.0	0.0	0.0		0.0	0.0	0.0	0.0	9.9
**Stage**	**PR**											
**Task No.**	**26**		**27**		**28**			**29**		**30**		**Overall Exclusion**
Exclusion (%)	11.1		12.1		2.4			4.8		**15.2**		15.3
Vision only (%)	4.1		4.1		1.1			0.0		4.1		4.1
Hearing only (%)	2.3		0.0		0.0			2.3		2.3		2.3
Thinking only (%)	4.4		1.4		1.4			3.2		4.4		5.0
Reach & dex only (%)	5.4		0.0		0.0			0.0		5.4		5.4
Mobility only (%)	0.0		9.9		0.0			0.0		9.9		9.9

**Note**: Numbers in bold correspond to the highest excluded tasks.

**Figure 4:**
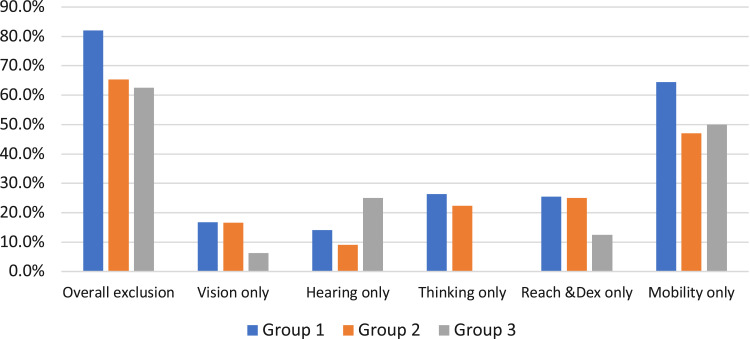
The exclusion number based on three different groups’ capabilities.

Figure 4 shows the results of the second way to estimate the exclusion, which presents the exclusion proportion based on the capabilities of three groups. Group 1: people with COPD within the British population database; Group 2: people with a respiratory issue within the British population database, and Group 3: people with COPD who comprised the 16 interviewed patients. Mobility exclusion was the highest of all the three groups. The overall exclusion number was very high, which indicates the importance of considering the capability-related needs of patients with COPD in order to improve access to care. Specifically, Table 7 shows the exclusion of people with COPD based on the DFS population database. About 82.1% of people with COPD were unlikely to access PR due to the service’s demand on their capabilities. The exclusion caused by mobility demand on patients was the highest among all the capabilities, accounting for 64.4%, which indicated the importance of understanding and considering the mobility demands that PR services place on patients. The exclusion of people with a respiratory issue when accessing PR was calculated as well (see Table 8). About 65.3% of people with a respiratory problem may be excluded from accessing the PR service due to their reduced capability. The exclusion made by the demand on patients’ mobility was the highest among those people who self-reported a respiration condition, making up 47.1%. Based on the capabilities of the 16 interviewed patients the proportion of people with COPD excluded from PR services was calculated to be approximately 62.5% (Table 9). Reduced mobility was the main factor that prevents people from accessing PR services. Although the exclusion number of thinking capability is 0, this does not illustrate that thinking capability does not affect people’s access to PR since the number of interviewed patients was small as a result of limited time and available resources.

**Table 7:** The Exclusion of People with COPD Based on the DFS Population Database

Category	V	H	Thinking					Reach & Dexterity								Mobility			Exclusion Calculation		
Capability and Related Exclusion	Vison	Hearing	Concentration	Remember	Literacy	Speaking Comprehension	Speaking	Lifting-Strength (Dom)	Dexterity (Dom)	Reach Forward and Up (Dom)	Reach Down (Dom)	Lifting-Strength (Non)	Dexterity (Non)	Reach Forward and Up (Dom)	Reach Down (Non)	Walking	Steps	Balance	People with COPD Within the Population Database (43,309,907)	Excluded Population of People with COPD	Percentage Excluded (People with COPD)
Capability demand	>	8	12	>	>	8	12	8	6	6	0	8	8	6	0	10	0	0			
Overall exclusion	12	8	12	12	12	8	12	8	6	6	0	8	8	6	0	10	0	0	180,786	148,471	82.1%
Vision only	12	0	0	0	0	0	0	0	0	0	0	0	0	0	0	0	0	0	180,786	30,405	16.8%
Hearing only	0	8	0	0	0	0	0	0	0	0	0	0	0	0	0	0	0	0	180,786	25,431	14.1%
Thinking only	0	0	12	12	12	8	12	0	0	0	0	0	0	0	0	0	0	0	180,786	47,562	26.3%
Reach & dex only	0	0	0	0	0	0	0	8	6	6	0	8	8	6	0	0	0	0	180,786	46,047	25.5%
Mobility only	0	0	0	0	0	0	0	0	0	0	0	0	0	0	0	10	0	0	180,786	116,346	64.4%

**Table 8:** The Exclusion of People with Respiratory Issue Based on the DFS Population Database

Category	V	H	Thinking					Reach & Dexterity								Mobility			Exclusion Calculation		
Capability and Related Exclusion	Vison	Hearing	Concentration	Remember	Literacy	Speaking Comprehension	Speaking	Lifting-Strength (Dom)	Dexterity (Dom)	Reach Forward and Up (Dom)	Reach Down (Dom)	Lifting-Strength (Non)	Dexterity (Non)	Reach Forward and Up (Dom)	Reach Down (Non)	Walking	Steps	Balance	People with Respiratory Issue Within the Population Database (43,309,907)	Excluded Population of People with Respiratory Issue	Percentage Excluded (People with Respiratory Issue)
Capability demand	>	8	12	>	>	8	12	8	6	6	0	8	8	6	0	10	0	0			
Overall exclusion	12	8	12	12	12	8	12	8	6	6	0	8	8	6	0	10	0	0	1,439,984	940,616	65.3%
Vision only	12	0	0	0	0	0	0	0	0	0	0	0	0	0	0	0	0	0	1,439,984	238,555	16.6%
Hearing only	0	8	0	0	0	0	0	0	0	0	0	0	0	0	0	0	0	0	1,439,984	131,207	9.1%
Thinking only	0	0	12	12	12	8	12	0	0	0	0	0	0	0	0	0	0	0	1,439,984	320,986	22.3%
Reach & dex only	0	0	0	0	0	0	0	8	6	6	0	8	8	6	0	0	0	0	1,439,984	360,084	25.0%
Mobility only	0	0	0	0	0	0	0	0	0	0	0	0	0	0	0	10	0	0	1,439,984	678,326	47.1%

**Table 9:** The Exclusion of People with COPD Based on the Interviewed Patients

Category	V	H	Thinking					Reach & Dexterity								Mobility			Exclusion Calculation		
Capability and Related Exclusion	Vison	Hearing	Concentration	Remember	Literacy	Speaking Comprehension	Speaking	Lifting-Strength (Dom)	Dexterity (Dom)	Reach Forward and Up (Dom)	Reach Down (Dom)	Lifting-Strength (Non)	Dexterity (Non)	Reach Forward and Up (Dom)	Reach Down (Non)	Walking	Steps	Balance	People with COPD based on the interviewed data	Excluded Population of People with COPD based on the interviewed patients	Percentage Excluded (People with COPD)
Capability demand	>	8	12	>	>	8	12	8	6	6	0	8	8	6	0	10	0	0			
Overall exclusion	12	8	12	12	12	8	12	8	6	6	0	8	8	6	0	10	0	0	16	10	62.5%
Vision only	12	0	0	0	0	0	0	0	0	0	0	0	0	0	0	0	0	0	16	1	6.3%
Hearing only	0	8	0	0	0	0	0	0	0	0	0	0	0	0	0	0	0	0	16	4	25%
Thinking only	0	0	12	12	12	8	12	0	0	0	0	0	0	0	0	0	0	0	16	0	0
Reach & dex only	0	0	0	0	0	0	0	8	6	6	0	8	8	6	0	0	0	0	16	2	12.5%
Mobility only	0	0	0	0	0	0	0	0	0	0	0	0	0	0	0	10	0	0	16	8	50.0%

It is important to emphasize that this Exclusion Calculator was based on a representative patient care journey which did, not cover all possibilities; even on the same care journey, different people had different capabilities to respond to the demands. Nevertheless, the exclusion calculation could indicate potential challenges that the demands of the service make on patients’ capabilities. Overall, the analysis in step 2 provides evidence that people’s access to PR services is potentially limited when the demands lie beyond their capabilities to respond.

### Identify Challenges (Step 3)

The main challenges for patients accessing PR services were divided into non-capability-related challenges and capability-related challenges (the left column of Table 10). In terms of capability-related challenges, a large proportion relates to COPD patients’ limited mobility, such as being unable to get to the assessment/PR class venue and having difficulty in climbing stairs or hills as their condition make them breathlessness. Other capability-related challenges include vision (the font size of the inhaler instructions is too small to read), hearing (cannot hear education class), language barriers and having other conditions such as arthritis and bad hips that affect PR class attendance. Non-capability-related challenges included perceptions of COPD (have never heard the term COPD or cannot accept the fact they have COPD), care-related challenges such as being unable to access proper information at each stage, long waiting times to start the PR program, and others such as emotional support and dependence on families/friends for transport. It is worth noting that some non-capability-related challenges may also influence patients’ capability and thus affect their access to PR. For example, the patients may feel anxious and depressed if they “have never heard the term COPD,” and thus they may not be able to concentrate (a part of thinking capability) on expressing their needs clearly (speaking, a part of thinking capability) and looking for help.

**Table 10:** The Main Challenges for Patients Accessing PR Services

Capability-related challenges	**1. Mobility**	
	Challenges Cannot get to the GP practice because of breathlessness Cannot get to the assessment/PR class venue Limited mobility in winter and bad weather (rain) Have difficulty in climbing stairs/hills Cannot walk long and need rest	Interpretation i–ii: It is likely to be difficult for patients with COPD to use transport to healthcare services. iii–v: Most people with COPD have limited mobility, in particular in bad weather/seasons and on certain roads, which affects their daily activities.
	**2. Other Capability (Vision, Hearing, Thinking, Reach&Dexterity)**	
	Challenges The font size on inhaler’s instruction is too small to read Vision declined due to COPD Some medication for COPD has side effects for vision Have hearing problems and need to wear a hearing aid Language barriers Other conditions such as arthritis and bad hips affect attending PR class	Interpretation i: The medication’s instruction needs to be well-designed to ensure patients with COPD can read it. ii–iii: The vision of patients with COPD may be affected by COPD medication. iv: Although hearing capability may not be affected by COPD, it somehow affects people’s access to PR such as make a call or listen to the education session. v: Some patients need interpreters’ help to access care. vii: Patient’s access PR may be affected by other conditions.
Non-capability-related challenges	**3. Perception About COPD**	
	Challenges Have never heard the term COPD Cannot accept the fact they have COPD	Interpretation i–ii: People not familiar with COPD may feel fear and not accept that they have COPD, and this may affect their thinking capability and mental health.
	**4. Care-Related**	
	Challenges Cannot access proper information in each stage GP/nurse just helps with one thing at a time The challenge of doing spirometry test Have no maintenance Have difficulty in quitting smoking Cannot commit to the PR class twice a week Long waiting time to get referred Long waiting time to start PR class	Interpretation i: Access to proper information is the key for people to self-manage their condition. ii–iv: The challenges originate from limited consultation time and health tests. v: Some patients need support to quit smoking. vi: Some patients may need the class tailored for them. vii–viii: Patients should be offered other alternative support during the waiting time.
	**5. Others**	
	Challenges Nobody to speak to Dependence on families/friends for transport Limited financial resources (cannot afford a scooter to commute)	Interpretation i: Patients need someone to talk to especially at the moment they are newly diagnosed. ii: Patients may rely on carer’s help, which means their attendance is also affected by the carer’s schedule. iii: Some patients may need financial support.

As shown on the right column of Table 10, the potential needs of patients were discerned from interpreting their challenges, which included transport services to PR venue, better guidance for using inhalers, a tailored PR program to match patients’ capability level, reassurance, and timely useful information and support in different stages to access PR services.

The main challenges for HCPs in engaging patients with PR were summarized in four categories: information, communication with patients, HCPs’ professional knowledge and other factors such as long waiting list for PR and influence from other patients (see Table 11 for details). The potential needs of HCPs in order to engage patients were also interpreted in accordance with the four categories (right column of Table 11), for example, face-to-face communication is vital to persuade patients to take up and attend PR. In addition, some HCP challenges may also suggest some potential patient needs, for example, the HCP challenge regarding communication with patients could suggest a patient need for face-to-face communication and for patients to be better understood by HCPs (category 2).

**Table 11:** The Main Challenges for HCPs to Engage Patients Access PR

**1. Information**		
Challenges Delay referring patients due to lack of patients’ contact information No leaflets available to offer to patients No up-to-date information for patients No up-to-date information about referral, eg, referral form	Interpretation i–iv: The efficiency of HCPs in obtaining information including patients’ information and treatment information affects how well they engage patients in attending PR.	
**2. Communication with Patients**		
Challenges Referrals are not done face-to-face by physios (some situations) No enough time to understand where patients are	Interpretation i–ii: Face-to-face communication is vital to persuade patients to take up and attend PR.	
**3**. **HCPs’ Professional Knowledge**		
Challenges Wrong diagnosis between asthma and COPD The skills for selling PR	Interpretation i: HCPs’ knowledge about distinguishing similar conditions. ii: The skills of HCPs to encourage patients to attend PR.	
**4. Others**		
Challenges Long waiting list causes patients to lose their initiative to attend PR Patients influence others’ attendance	Interpretation i: It is challenging to keep patients motivated to attend PR. ii: Patient’s attendance is affected by other participants.	

Furthermore, patients’ expectations and their needs from the HCPs perspective were summarized. Patients' expectations were: i) to have more information about treatment; ii) to be offered other formats of PR such as a TV program; iii) to see their own GP rather than a GP who does not know them; iv) to be referred to PR earlier; v) to have someone to talk to about their condition. Patient’s needs from the HCPs perspective were: i) reassurance and understanding when informed of their diagnosis; ii) attention to psychological health; iii) tailored PR service to meet patients’ needs if possible; iv) transport help; and v) referral at early stage.

### Propose Recommendations (Step 4)

Three themes of recommendations are suggested below to increase patients’ access to PR (Figure 3):

- Reassurance: patients should be offered an overall picture of the COPD care journey and should engage with decision-making, thus giving them a sense of control of their condition.
- Information: timely useful information should be provided to patients at each stage.
- Support: flexible and timely treatment options should be offered to patients, for example, other formats such as videos could be available for patients who are not able to get to the class (transport).

The Initial Recommendations were formulated to provide patients with reassurance, information and support in different stages of their COPD care journey (Table 12). For patients with a hearing problem, HCPs may need pay more attention to them especially during the education session. Similarly, in the COPD diagnosis stage, HCPs should reassure patients through face-to-face communication, provide patients with useful information to understand COPD and avoid communicating their diagnosis by letter. As a possible result, patients are more likely to become involved in self-managing their condition and actively in attending PR. Also, it is important to offer multiple treatment options to patients to make them feel supported, in particular, promoting PR to patients.

**Table 12:** Initial Recommendations for Improving Patients Access to PR Services

Stage	Recommendations
COPD Diagnosis	Reassure patients by offering them proper information to understand COPD conditions. (Thinking) Offer multiple treatment options to patients to make them feel supported, in particular, advertising PR to patients.
Regular review	Remind patients to have their regular reviews for COPD. (Thinking) Provide patients with information and support after review if needed.
Referral to PR	Offer patients PR and provide contact information to patients for them to self-check. (Thinking) Send invitation for PR to patients in a timely manner.
Assessment to PR	Provide patients with clear navigation instructions to get to the assessment venue. (Vision) Remind patients to bring documents especial medication list when coming for assessment. (Thinking) Sell PR to patients and try to meet patients’ needs.
PR	Providing patients with transportation if they have difficulty in getting to PR. (Mobility) Tailor the PR class to patients if needed. (Reach & Dexterity, Mobility) Pay attention to patients who have a hearing impairment or declined thinking. (Hearing) Encourage patients to do exercises at home. Offer support if patients need it.

In terms of the results of evaluating the recommendations, two focus groups with patients (n=4, and n=5) gave very helpful comments to improve the COPD reminder. For example, the initial COPD recommendations only had five scenarios which were based on the five main stages of the COPD primary care pathway. However, during the first focus group meeting, patients raised questions about after PR, “what happens next? What is the future? I have gone through all the five stages of COPD.” As a consequence, another scenario, ie, “6. Next” has been added to provide patients with more information and support since proper access information can reassure patients, which may influence patients’ concentration. 10 HCPs participated the survey and all of them thought the reminder was easy to understand, and most of them (8/10) agreed that it is good to use patients’ care journey to remind them manage their care. The critical feedbacks were around the contents (elements) that should be included in the reminder and the effectiveness of using the “Your COPD Care Reminder”. For example, one HCP mentioned, “a reminder that ongoing exercise after PR is essential”, which is a very useful comment and has been added in “4. PR programme”. The third focus groups with patients (n=8) reviewed positive feedback. Patients who participated the evaluation thought it was a convenient and portable tool for them to know their situation and manage their COPD. In particular, a patient who was newly diagnosed with COPD and had not been referred to PR spoke highly of the reminder. Meanwhile, some useful comments were collected, for example, introducing the Breathe Easy Support Group to patients in scenario “6. Next” to help patients find groups and support.

**Figure 5:**
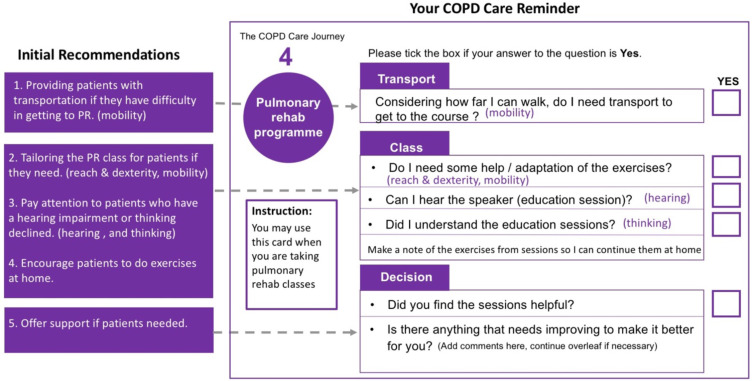
The PR programme scenario of the COPD care reminder.

Finally, the initial recommendations were integrated into seven cards, which include six scenarios and a set of questions (called Your COPD Care Reminder, see Figure S2). The recommendations encourage patients to consider their potential needs along their COPD care journey, in particular, those needs that are caused by their reduced capabilities when trying to access PR. Figure 5 shows the card in the PR program scenario, where some key issues relating to patients’ capabilities were highlighted to encourage patients to reflect on their situation. For instance, patients may consider their Reach & Dexterity, and mobility to ask themselves, “Do I need some help/adaptation of the exercises?” In this way, PR physiotherapists would be better aware of the needs to tailor exercises and patients may be also self-aware of this need. “Your COPD Care Reminder” can not only acts as a reminder to patients, but also as an interactive tool for patients to actively communicate with HCPs.

## Discussion

### Main Findings

This is the first study that used an Inclusive Design approach to explore how patients’ capabilities influence their uptake of PR. It aims to offer healthcare researchers and providers another way to consider the capability-related needs of people accessing PR services. The study demonstrated that COPD patients’ capability level may affect their uptake of PR services. It was estimated in step 2 that at least 15.6% of the British population and 82.1% of people with COPD are likely to be excluded from PR services based on Mrs. Smith’s scenario. Although the estimation was based on one patient persona, the results can still indicate the importance of considering patients’ capability in improving the utilization of PR services. The challenges identified in step 3 also demonstrated that, besides the barriers discovered in previous studies,^27^ there are also a number of barriers related to patients’ capabilities, for example, other conditions-arthritis and bad hips affect COPD patients attending PR class. Therefore, it is helpful to estimate COPD patients’ capability and minimize the capability-related barriers for them to access PR.

### Strengths

The study also demonstrated that an Inclusive Design approach can be used to analyze the accessibility of PR services. The inclusive approach to PR service design comprises four steps, and is a user-focused system design process, rather than a single tool or method. In Step 1 mapping care journeys, Inclusive Design could help researchers and care providers to understand patients’ tasks along with their care journey and lay the foundation for improving patients’ experiences. Applying the Inclusive Design approach to improving the PR service requires a clear picture of how the PR service is delivered and how patients access the current service system. The relevant methods and tools include data collection methods such as interviews and surveys with COPD patients and HCPs (including care providers and commissioners) to share their experiences about delivering and receiving the current PR service respectively.

In Step 2 estimate exclusion, Inclusive Design can be used to estimate a service’s demands on patients and the level of potential exclusion. The Inclusive Design tool, Exclusion Calculator, plays two roles in this step: i) the pre-defined scales within the tool are the criteria used to estimate the service’s demand on patients; ii) the database of the British population’s capabilities within the tool can be used to estimate the service’s exclusion based on the rated demand scales. It is worth noting that the database within the Exclusion Calculator can be adjusted depending on the requirement. The current population database can be filtered to specific groups (people with COPD, people with respiratory issue) or changed to other available databases to estimate the service exclusion. Also, two “aided tools” for Inclusive Design, the persona and scenario, are useful in defining the scope for estimate exclusion.

In Step 3 identify challenges, Inclusive Design could provide indicators of potential challenges and needs for patients to access PR. The main challenges for COPD patients accessing PR services were indicated by two methods: one is by consulting the exclusion data in step 2, and the other is by further analyzing the data in step 1 which could extract the challenges and needs of patients. The identified challenges can be translated into potential needs. For example, patients may have difficulty in getting to the PR assessment venue which is relatively far and unfamiliar to them. This challenge can be interpreted as patients needing transport to support them to access the PR service.

In Step 4 propose recommendations, Inclusive Design could help suggest recommendations to address patients’ capability-related needs along their care journey and thus offer an inclusive experience for patients accessing PR. The recommendations could be validated and refined together with patients and HCPs through interviews, focus groups or surveys. These recommendations could be used in different formats for patients, HCPs and healthcare providers depending on use or requirements. For example, “Your COPD Care Reminder” can not only acts as a reminder to patients, but also as an interactive tool for patients to actively communicate with HCPs. A couple of questions within “Your COPD Care Reminder” were designed to remind patients to consider whether their capabilities meet the demands of some tasks along their care journeys in different scenarios. Patients are recommended to request help if they are unable to do some tasks. Those questions can also help HCPs to understand the potential needs of patients. For example, in scenario 4. Pulmonary rehab program (see Figure 5), patients are required to think of a series of questions about their capability to attend class. The question “Can I hear the speaker?” relates to patients’ hearing. As a result, patients could have a better understanding about their situation and request more help if needed, which could also give them a feeling of control over their condition. The question can also raise physiotherapists’ awareness that some patients may have hearing problems, so the speakers may provide some printed materials as a supplement. In this sense, Inclusive Design could improve patients’ experience in accessing PR services.

“Your COPD care reminder” can be printed and disseminated in GP practices, hospitals, patient support groups and other places that COPD patients may access. Some recommendations such as transport services may require support from PR service providers and/or health authorities. When using this Inclusive Design approach, three groups of people should be involved: care providers, HCPs and healthcare researchers should be involved as they are familiar with healthcare services system; Inclusive Design experts or people who have some design knowledge should also be invited into the team; it is also vital to involve patients who are the service users.

### Limitations

There are mainly two limitations of conducting this study. One is that the patients that were involved in interviews may not be representative of all patients, since for example, it was proved to be difficult to recruit patients with end-stage COPD and disabled people.^44^ We did ensure a full range of capability loss is covered by the samples and some participants are disabled, there are still some capability-related needs for these patient categories might not be covered. Mrs. Smith’s scenario is one of the most representative care journeys and the challenges she faced may not cover all the possibilities of COPD patients. Also, the population database used for estimating excluded COPD patients was limited to the data from Disability Follow-up to the Family Resources Survey in the UK, although it remains the most holistic source of UK data. The findings we presented may be restricted to the situation in the UK, and future research can build cases in other countries to compare and contrast the findings between this study and future work.

### Study Implications

Further work could focus on implementing these recommendations to alter existing PR service routines and evaluate whether access is subsequently improved. Also, further development of the Inclusive Design approach could explore ways to make it as easy as possible for healthcare providers and researchers to use, including developing tools and guidance for implementing Inclusive Design methods. In addition, cognitive dysfunction (eg, anxiety and depression) that also affects COPD patients’ access to PR could be an extension of this study, if a more comprehensive population database that includes psychology-related capability data is available.

## Conclusion

PR is a highly recommended intervention for people with COPD. However, the uptake and attendance of PR is extremely low in the UK. In this study, we have demonstrated that failure to take patients’ capability needs into account in the delivery of PR may act as a barrier to the uptake and attendance of PR in the UK. It also highlights the importance of providing COPD patients with proper information and flexible treatment options in reassuring patients to uptake and attend PR. The “Your COPD Care Reminder” developed in this study sets an example for how to engage patients in addressing their capability-related needs, which can also be us This study has further demonstrated that the application of Inclusive Design to health services is possible and the tools applied can make a useful contribution to understanding PR service provision and hence service improvement. The Inclusive Design approach not only helps care providers, HCPs and healthcare researchers to consider patients’ capabilities when designing healthcare services, but it also raises patients’ awareness of their own capability-related needs and need to actively request help.
